# Overview of the VA Quality Enhancement Research Initiative (QUERI) and QUERI theme articles: QUERI Series

**DOI:** 10.1186/1748-5908-3-8

**Published:** 2008-02-15

**Authors:** Cheryl B Stetler, Brian S Mittman, Joseph Francis

**Affiliations:** 1 Independent Consultant, Amherst, Massachusetts, USA; 2 VA Center for the Study of Healthcare Provider Behavior, Veterans Affairs Greater Los Angeles Healthcare System, Los Angeles, California, USA; 3 Office of Research and Development, Department of Veterans Affairs, Washington, DC, USA

## Abstract

**Background:**

Continuing challenges to timely adoption of evidence-based clinical practices in healthcare have generated intense interest in the development and application of new implementation methods and frameworks. These challenges led the United States (U.S.) Department of Veterans Affairs (VA) to create the Quality Enhancement Research Initiative (QUERI) in the late 1990s. QUERI's purpose was to harness VA's health services research expertise and resources in an ongoing system-wide effort to improve the performance of the VA healthcare system and, thus, quality of care for veterans. QUERI in turn created a systematic means of involving VA researchers both in enhancing VA healthcare quality, by implementing evidence-based practices, and in contributing to the continuing development of implementation science.

The efforts of VA researchers to improve healthcare delivery practices through QUERI and related initiatives are documented in a growing body of literature. The scientific frameworks and methodological approaches developed and employed by QUERI are less well described. A QUERI Series of articles in *Implementation Science *will illustrate many of these QUERI tools. This *Overview *article introduces both QUERI and the Series.

**Methods:**

The *Overview *briefly explains the purpose and context of the QUERI Program. It then describes the following: the key operational structure of QUERI Centers, guiding frameworks designed to enhance implementation and related research, QUERI's progress and promise to date, and the Series' general content. QUERI's frameworks include a core set of steps for diagnosing and closing quality gaps and, simultaneously, advancing implementation science. Throughout the paper, the envisioned involvement and activities of VA researchers within QUERI Centers also are highlighted. The Series is then described, illustrating the use of QUERI frameworks and other tools designed to respond to implementation challenges.

**Conclusion:**

QUERI's simultaneous pursuit of improvement and research goals within a large healthcare system may be unique. However, descriptions of this still-evolving effort, including its conceptual frameworks, methodological approaches, and enabling processes, should have applicability to implementation researchers in a range of health care settings. Thus, the *Series *is offered as a resource for other implementation research programs and researchers pursuing common goals in improving care and developing the field of implementation science.

## Background

Improving the quality and performance of healthcare delivery systems represents one of the most significant challenges facing government and society in the U.S. and internationally. A promising strategy for improving healthcare quality is the systematic implementation of research findings and related practices known to generate better outcomes than prevailing practices. Unfortunately, barriers to successful implementation of effective practices are considerable and not fully understood; and reliable, effective strategies to facilitate implementation, particularly on a routine basis, are underutilized.

In 1998, the U.S. Department of Veterans Affairs' (VA) Quality Enhancement Research Initiative (QUERI) was established to improve the quality of VA healthcare through the use of research-derived best practices. During QUERI's initial development, new organizational structures, roles and procedures were established to facilitate active participation and collaboration among a large, multi-disciplinary group of VA researchers, managers and other key stakeholders. For their part, QUERI researchers employed concepts and approaches from what has become known as implementation science, also encompassed in the literature by terms such as knowledge utilization, knowledge translation, and knowledge transfer [1,2]. They quickly discovered challenges both to implementation and the effective conduct of implementation research – challenges that continue to be encountered today. These included the need for new research designs, methods and instruments [3-6];better reporting templates [6,7]; and increasing recognition that implementation is often incredibly complex [7]. Additionally, it was noted that "while there are several theoretical or conceptual models to pursue for guidance, there remain [ed] a need for the literature to document... [related] field-level successes and failures [p. 173, [7]]."

Given the limited guidance available to implementation researchers in the Program's early years, QUERI researchers tried not only well-known interventions and models from various fields but also innovative approaches [8-11]. Consideration of such diverse sources enabled QUERI researchers to better understand and address methodological issues and barriers to adoption and sustainability. Resultant QUERI implementation projects, with their "field-level successes and failures" [7] related to the use of such approaches, have produced a myriad of insights and refinements as described in the QUERI *Series*.

This *Overview *article introduces the QUERI Program and its guiding frameworks. The *Overview *also summarizes QUERI's progress and promise. Finally, it introduces the QUERI *Series*, which presents and illustrates QUERI's implementation research frameworks, as well as a range of other conceptual and practical tools designed to address the challenges of implementation and related research. Overall, this content offers insights for other health systems and researchers seeking to effectively apply research to improve the care of patients.

## The QUERI program

QUERI was created within the context of an internationally recognized transformation of the VA's healthcare delivery system. This transformation had at its core a "quality improvement lens" [12,13], and involved a major redesign of organizational structures and policies, including implementation of innovative information technology and a new performance management/accountability program [14]. Within this overall transformation, QUERI was established inside the Health Services and Research Development (HSR&D) arm of the VA to "purposely link research activities (which generate scientific evidence) to clinical care in as close to real time as possible [p. I-14, [15]]," in order to enhance the "rapid adoption of best clinical practices and improvement in patient outcomes [p. I-14, [15]]."

Even prior to QUERI, the VA had recognized the value of research to improving patient care by supporting an intramural research program whose statutory mission was to enhance the health of veterans [14]. By embedding investigators within a fully integrated delivery system with a stable patient population and robust electronic health records, VA had unparalleled opportunities to translate clinical questions into research studies and research findings into clinical actions. For instance, since 1946, VA has conducted multi-site clinical trials and has maintained a network of regional support centers that facilitate the evaluation of both standard and novel therapies [16]. VA's ability to conduct clinical trials of practical significance to the population it serves was well recognized as a resource that could generate evidence "ripe" for implementation [17]. In fact, the VA has served to generate a good deal of the clinical evidence currently considered "best practice," such as the routine use of aspirin for acute coronary artery syndromes [18].

Additional VA work, primarily in the field of health services research, laid further groundwork for implementation by using electronic administrative and clinical data to identify both variations in practice patterns across VA facilities and the considerable gap between ideal and actual clinical practice. VA work also had identified the reality that doing the right research and disseminating its findings was insufficient to transform health care [19]. In response, QUERI was created to generate research-driven initiatives to directly and rapidly achieve quality improvements, including measurable progress in system performance and health-related outcomes. Although "research-driven," QUERI activity was in reality to occur within the context of collaboration and cooperation among researchers, policy makers, and local leaders within VA's decentralized, geographically-based clinical delivery networks.

QUERI has been described previously in detail [20,21] and findings from various QUERI projects have been published [8,22-24]. To date, however, the implementation science frameworks and methodological approaches developed and employed by QUERI have not been well documented.

## QUERI Centers and guiding frameworks

The core QUERI approach was designed by key VA health system and research leaders exploring new strategies for achieving rapid VA quality improvements [personal communication, J Demakis]. The original design included the need to involve researchers more directly and systematically in promoting guideline-based practice and reducing the gaps between routine practice and the best available evidence. Through QUERI, VA leadership envisioned a proactive, interactive and multi-faceted implementation role for health services researchers in the context of close collaboration between research, quality improvement (QI) and clinical leadership.

Key elements of the QUERI Program evolved over time. These include a set of disease or problem-focused QUERI Centers, a core set of program-wide goals, and a complex 6-step framework, or "process," that guides each Center's activities. A QUERI Center is an organizational structure that provides dedicated infrastructure support, including a core team consisting of a research coordinator, clinical coordinator and implementation research coordinator. This core team shares operational responsibility to implement the QUERI process (described below). QUERI Centers may be housed within a single VA facility or organized "virtually" across several sites, but each is tasked with system-wide, rather than solely local responsibility. These duties include: 1) establishing a network of affiliated researchers, 2) making contacts with local and national clinical and policy leaders, and 3) directing the work of the Center strategically by focusing on system-wide priorities for improvement [20,21].

Each QUERI Center focuses on a specific patient population or condition that has been identified by VA leadership as a high-risk/high-volume priority for the health care system. There are currently nine such Centers (Chronic Heart Failure, Diabetes, HIV/Hepatitis, Ischemic Heart Disease, Mental Health, Polytrauma/Blast-Related Injuries, Spinal Cord Injury, Stroke, and Substance Use Disorders) [25]. Each QUERI Center is guided by a multidisciplinary Executive Committee comprised of experts and key stakeholders. This group helps their respective Center develop strategic plans to prioritize and initiate activities addressing their designated clinical condition. Overall, each QUERI Center aims to create the following:

### 1. A structured program of implementation research

This aim focuses on implementing evidence-based "best practices" and improving current patient and system outcomes for their patient population, as close to real time as possible, through the use of active, evidence-based implementation approaches.

### 2. New implementation research findings and insights

This aim focuses on the implementation process both in general and relative to a Center's specific patient population in order to: a) continually strengthen VA's ability to accelerate routine, rapid uptake and spread of evidence-based practices throughout the health care system, and b) contribute to the field of implementation science for the benefit of implementation stakeholders within and outside the VA.

With those aims in mind, QUERI Centers are responsible for monitoring, understanding, evaluating, and acting upon both emerging clinical research findings and implementation research findings that provide strategies for improving their target populations' care and outcomes. Therefore, QUERI researchers are involved in both investigating a broad spectrum of implementation issues and, simultaneously, pursuing significant improvements within participating study sites – and, if appropriate, working to subsequently spread improvements across the system and to study that aspect of implementation as well. Consistent with the overall VA transformation, QUERI Centers are held accountable for their performance related to these goals.

The research activities of QUERI Centers include a broad range of implementation projects, as well as variation and outcomes studies to document and understand current clinical practices and quality gaps. QUERI Centers also work to identify, develop and/or refine implementation approaches (e.g., individual adoption interventions or measurement tools) that are then incorporated into implementation projects. All of this activity is guided by a QUERI framework or core 6-step process that has evolved since QUERI's inception in 1998. This core conceptualization of the implementation process offers an explicit series of steps for diagnosing and closing quality gaps, and, simultaneously, advancing knowledge in implementation science. This core process consists of the following steps:

1) Identifying high-risk/high-volume diseases or problems,

2) Identifying best practices,

3) Defining existing practice patterns and outcomes across the VA and current variation from best practices,

4) Identifying and implementing interventions to promote best practices,

5) Documenting that best practices improve outcomes, and

6) Documenting that outcomes are associated with improved health-related quality of life.

Steps 4 through 6 usually co-occur within individual implementation projects. Details regarding these steps, which have evolved and been clarified over time, are provided in Table 1. It should be noted that two additional steps have been added to the core process: 1) preliminary efficacy/effectiveness studies of highly promising clinical/delivery system interventions, at times needed as pre-implementation work; and 2) development and/or evaluation of needed tools and measurements.

**Table 1:** Summary and description of expanded six-step QUERI process model

**CORE STEPS**

**Step 1: Select conditions per patient populations associated with high risk of disease and/or disability and/or burden of illness for veterans**
1A. Identify and prioritize (via a formal ranking procedure)
1B. Identify high-priority clinical practices and outcomes within a selected condition
▪ *Overall conditions addressed by QUERI Coordinating Centers are selected by the VHA [Veterans Health Administration; also referred to as VA in this Series] and national QUERI leadership. QUERI Center Executive Committee then assigns priorities to specific sub-topics within each clinical area selected to provide the greatest possible impact on veteran health*.
▪ *QUERI groups seek opportunities for collaboration on overlapping priorities, such as relevant coexisting diagnoses (e.g., mental illness and substance use disorder)*.
▪ *Epidemiological and outcomes studies may be conducted or, if available, used to facilitate decision making*.
**Step 2: Identify evidence-based guidelines, recommendations and best practices**
2A. Identify evidence-based clinical practice guidelines
2B. Identify evidence-based clinical recommendations
2C. Identify evidence-based clinical practices
▪ *Can include systematic reviews and/or a consensus process*
**Step 3: Measure and diagnose quality and performance gaps**
3A. Measure existing practice patterns and outcomes across VA and identify variations from evidence-based practices ("quality/performance gaps")
3B. Identify determinants of current practices
3C. Diagnose quality/performance gaps
3D. Identify barriers and facilitators to improvement
▪ *Includes variations studies to a) measure care processes related to clinical conditions and related deviations from best practices, and b) explain various influences on practices*.
▪ *Studies focus on general, VA-wide gaps relative to a targeted condition or issue*.
**Step 4: Implement improvement programs**
4A. Identify improvement/implementation strategies, programs and program components or tools
4B. Develop or adapt improvement/implementation strategies, programs and program components or tools
4C. Implement improvement/implementation strategies/programs to address quality gaps
▪ *Requires literature searches for evidence-based implementation interventions, change strategies and related tools*.
▪ *Includes development and evaluation of implementation or practice support toolkits, such as educational materials or clinical reminder content*.
▪ *Researchers expected to consider relevant methodological approaches, e.g., a conceptual framework, an appropriate study design and facilitation [11]*.
**Step 5/6: Evaluate improvement programs**
5. Assess improvement program feasibility, implementation and impacts on patient, family and healthcare system processes and outcomes
6. Assess improvement program impacts on health related quality of life (HRQOL)
▪ *Should consider both formative and summative evaluation*.
▪ *As part of formative evaluation [FE], would include a developmental-stage local diagnostic analysis to affirm generically identified barriers in study sites; would also consider other FE stages [9]*.
▪ *Should consider a cost- or business case analysis*.
**SUPPLEMENTAL RESEARCH ACTIVITIES**

**Step M: Develop measures, methods and data resources**
M1. Develop, refine and validate patient registries and databases documenting healthcare organizational features, clinical practices and utilization, and outcomes.
M2. Develop and/or evaluate case-finding and screening tools.
M3. Develop and/or evaluate measures of healthcare structures, processes and outcomes.
**Step C: Develop clinical evidence**
C1. Develop and evaluate evidence-based clinical practices and recommendations (clinical research).
C2. Develop and evaluate evidence-based health services interventions (health services research).
▪ *Step M and C projects are considered to be outside the core QUERI process, although they support implementation research. Such projects are generally funded through regular VHA or external clinical science and health services research funding programs*.

The expanded 6-step process also has been supplemented with additional frameworks and other implementation tools over time. These include various documents that provide general guidance for enacting and enhancing the usefulness of the 6-step process as well as a comprehensive glossary to facilitate communication and consistency within QUERI (See Additional File 1 Key QUERI Definitions). Some of these tools have been adopted or refined from prior research, although given QUERI's early start (1998), relevant guidance was frequently not available or was insufficient to meet the pragmatic needs of QUERI researchers. Three tools, designed for Step 4 of the process and highlighted below, are particularly central to QUERI and are described or illustrated in various *Series *articles:

▪ A *4-phase pipeline framework *that facilitates the expected programmatic progression of QUERI Center implementation activity. Based on previously-developed phase models, the QUERI 4-phase framework describes a sequence of implementation projects from initial feasibility assessment to national roll-out. As noted above, targeted pre-implementation activity (e.g., critical measurement development or affirmation of promising interventions) also may occur within a QUERI Center to feed and enhance this pipeline. See Table 2 for more detail.

**Table 2:** QUERI phases of implementation projects/QUERI pipeline

Phase 1: Pilot project to develop/refine an improvement/implementation program and assess basic feasibility:
◆ Small scale study within a single clinic or facility
◆ Used with a substantiated clinical or delivery best practice
◆ Identifies potential issues relative to routine integration of best practice such as acceptability of the recommendation, process barriers, and needed toolkit elements
Phase 2: Small clinical trials to further refine and evaluate an improvement/implementation program
◆ Relatively modest but multi-site evaluation (e.g., 4-6 facilities within one or two VA regions)
◆ Conducted within a formal research and evaluation framework, e.g., an experimental design. Usually is a hybrid design, i.e., a traditional intervention design plus a descriptive formative evaluation [9]
◆ Requires active research team support and involvement, plus modest real-time refinements to maximize the likelihood of success and to study the process for replication requirements
◆ Enables refinement before larger-scale implementation
Phase 3: "Regional roll-out" projects
◆ Test of large-scale adoption program prior to full VA implementation with 10-20 facilities in 3-5 VA regions
◆ Decreased research team support at local sites and greater involvement of stakeholders, both nationally and locally
◆ Should require less need for real-time refinements of the implementation strategy
◆ Preparation for hand-off at national level
Phase 4: "National roll-out" effort
◆ Implementation of a tested, refined strategy throughout the VA
◆ Existing operations or designated leadership entity deliver the program
◆ Research team support as determined per Phase 3 evaluation
◆ Concurrent and ongoing evaluation, per methodology determined/refined in Phase 3

▪ A *Service Directed Project *(SDP) program and template involving a) an innovative funding mechanism supported by clinical operations funds rather than research monies {an exceptional arrangement within the VA} and b) a set of explicit study design recommendations. The design template has encouraged researchers to employ a more active, hands-on approach to implementation and its study [26] (Also see Additional File 2 VA QUERI Service Directed Projects: Proposal Review). More specifically, SDPs encourage the following: explicit exploration of the black box of implementation; optimal implementation of the change intervention during the study to enhance successful "uptake" and outcomes improvement in the targeted study sites – or at least assessment of the potential to do so; and development and clear articulation of a replicable implementation program.

▪ *An approach to QUERI proposal review *(closely linked to the SDP concept), which includes a uniquely crafted process for peer-review of scientific and policy/practice merit. This process incorporates unique considerations of implementation science along with more traditional methodological criteria. Using this approach, review committees are constituted to include the appropriate range of scientific expertise along with clinical program leaders that can speak to relevant policy and practice issues. These issues include the importance of the implementation target relative to other organizational priorities, the business case for the proposed implementation program, and the likelihood for long-term sustainability after project completion. Additional file 2 reproduces critical aspects of a checklist provided to QUERI reviewers to emphasize implementation-oriented criteria (See Additional File 2* VA QUERI Service Directed Projects: Proposal Review*).

## Progress and promise

Seven of the QUERI Centers have been in existence for several years, and two were established more recently. Each Center has a program of research encompassing QUERI Steps 1 through 5/6. Development of a full portfolio of implementation-related research for each QUERI Center has taken time, given the following:

▪ The need for the QUERI Program to develop an understanding of its mission and to develop effective practice-oriented research activities and approaches for QUERI researchers and other stakeholders.

▪ The need for many QUERI researchers to obtain grounding in the field of implementation science and related disciplines (e.g., organizational science and anthropology), as well as in unfamiliar methods (e.g., formative evaluation and qualitative methods).

▪ Funding, proposal review and ethics review (Institutional Review Board) timelines.

▪ The need to develop frameworks and other tools to guide the envisioned implementation activity and research.

To develop and implement a comprehensive strategic plan, each QUERI Center has established a rich set of collaborative relationships involving numerous national and regional (VA and non-VA) stakeholder groups. These include, for example, VA's Office of Quality and Performance (responsible for VA's extensive performance measurement and feedback system) and directors of VA's 21 regional healthcare networks, each within a defined geographical area of the US. These networks comprise VA's full spectrum of healthcare delivery facilities, including primary, tertiary, long-term, and other specialized care. An additional file illustrates partnerships for one QUERI Center (See Additional File 3 Key Stroke QUERI Collaborators Diagram). These relationships form the basis for partnering with key policy and clinical stakeholders, recognized as critical to making implementation a "system" rather than solely a "research" or "researcher" issue.

Although QUERI should still be considered a work in progress, much like the overall field of implementation science, the results of QUERI efforts to study and improve VA healthcare delivery practices are documented in a growing body of journal articles and reports [27-32]. In particular, QUERI-related publications and presentations reflect the steps of the QUERI process, as follows (see Table 1): pre-implementation intervention studies establishing best practices or measurement tools, per Steps M and C [33-40]; research and related activities relevant to QUERI Steps 1 to 3, e.g., regarding best and current practices [41-47]; activity relevant to QUERI Step 4/5/6 projects, including implementation trials and studies employing non-experimental designs [8,22,48-55]; and specific outcomes of overall QUERI efforts [19]. In terms of the latter, for example, the following have been demonstrated: improvement in evidence-based alcohol screening; expansion of the number of methadone clinics within VA for veterans with opioid dependence; increase in the proportion of veterans with spinal cord injury receiving influenza and pneumococcal vaccinations; and a change in a prevailing performance measure to improve eye care in diabetics by focusing policy on the needs of veterans at high risk for blindness [23,24,50,55,56]. Finally, a large regional-level demonstration project, guided by the Mental Health QUERI Center, is accumulating knowledge and laying the groundwork and support for the national spread and sustainability of evidence-based collaborative care for depression. This effort has achieved an unprecedented level of researcher involvement and linkage to stakeholders within an implementation project and has already led to a nationally-supported program to enhance primary care/mental health collaboration across the VA healthcare system [48,57].

## QUERI Series

The implementation approaches underlying QUERI successes are just beginning to be documented and disseminated, particularly in relation to specific Step 4 demonstration projects and general contributions to implementation science. Some of these build upon the work of others in the field of implementation science, while others reflect innovations developed by QUERI. For example, previously published articles present a refined view of formative evaluation within implementation [9]; the interconnection between theories, models, strategies, other tools, and planning [10]; and an exploration of the concept of facilitation within the PARIHS (Promoting Action on Research Implementation in Health Systems) framework [11]. However, the general use of such approaches in QUERI has not, up to now, been illustrated, and other QUERI-related frameworks have yet to be explicated. Describing these tools and their integration within QUERI projects is relevant for the field of implementation science, other healthcare delivery systems, and researchers interested in replicating or exploring QUERI's improvement model and insights. The purpose of this *QUERI Series *for *Implementation Science *is to document and share this information in the context of its use within QUERI Centers' programmatic implementation research (Tables 1 and 2). Thus, the *Series *articles demonstrate how QUERI conceptualizes, designs, enables and conducts implementation research, and, consequently, how it develops new insights into implementation science.

The QUERI *Series *opens with this *Overview*, describing the QUERI program, its operational QUERI Centers, and its key, overarching *Frameworks*. These articles are followed by papers that primarily represent QUERI Centers' work and then a set of responsive commentaries. The former papers focus on a range of QUERI implementation research approaches, implementation study issues and needs, implementation barriers and enabling factors at both micro and macro levels, and illustrative cases demonstrating the use of various implementation tools including the core 6-step process. Cumulatively, this *Series *describes a broad array of implementation research challenges, as well as potential approaches explored by QUERI researchers to meet those challenges. The commentaries at the end of the *Series *provide reflections on the potential value of QUERI and its related approaches from the perspective of both VA (non-QUERI) leadership and non-VA stakeholders.

## Conclusion

Development and use of QUERI's implementation science frameworks and methodological approaches have generated excitement and frustration, enjoyed successes and encountered barriers, and continuously enhanced progress in the understanding of implementation concepts and strategies. Insights gained to date regarding implementation science are now being incorporated into the next phases of QUERI Center programmatic research. The full potential and influence of QUERI should emerge over the next few years a) as all Centers continue to progress from early pilots and demonstration projects to large-scale regional trials and b) as the organizational template for national implementation under development by the Mental Health QUERI Center is evaluated and replicated by others.

VA and QUERI are not unique in their efforts to employ research-based approaches to accelerate routine implementation of evidence into practice within an integrated delivery system, although QUERI's simultaneous pursuit of improvement and implementation research goals may be unique. However, the richly detailed descriptions of this still-evolving effort and its frameworks, other tools, and enabling processes should have applicability to implementation researchers as well as health system leaders. With this *Series*, publications appearing elsewhere, and considerable work-in-progress, QUERI is pleased to share its evidence-based implementation experiences and evolving conceptual knowledge with colleagues also engaged on the journey to close the gaps in implementation knowledge and clinical practice.

## Competing interests

Brian Mittman is Co-Editor-in-Chief of *Implementation Science*; Joe Francis is a member of the Editorial Board. All editorial decisions regarding this article and all subsequent articles in the QUERI Series were made independently by Martin Eccles, Co-Editor-in-Chief, and Ian Graham, a member of the Editorial Board serving as special co-editor of the Series. The articles in the QUERI Series describe implementation research conducted within the Health Services Research and Development (HSR&D) Service of the U.S. Department of Veterans Affairs. VA HSR&D provides in-kind support for the journal *Implementation Science*, including salary support for Brian Mittman and support for editorial and copy editing services. VA HSR&D staff played no role in editorial decisions for the QUERI Series manuscripts. The primary author (CBS) has worked as a QUERI consultant for several years.

## Authors' contributions

CBS drafted the initial form and all revisions of this manuscript. BSM has read and drafted substantial refinements, and JF has provided input, feedback and refinements to the initial and final versions. All authors agreed to the final manuscript.

## Supplementary Material

Additional file 1 *Key QUERI Definitions*. A glossary developed to facilitate communication and consistency within QUERI. Click here for file

Additional file 2 *VA QUERI Service Directed Projects: Proposal Review*. Critical aspects of a proposal review checklist developed to emphasize implementation-oriented criteria. Click here for file

Additional file 3 *Key Stroke QUERI Collaborators Diagram*. Sample of the type of partnerships established by a QUERI Center. Click here for file
