# Supplementary material for: Overview of the VA Quality Enhancement Research Initiative (QUERI) and QUERI theme articles: QUERI Series
Source: Implement Sci. 2008 Feb 15;3:8. doi: 10.1186/1748-5908-3-8 (PMC2289837; doi:10.1186/1748-5908-3-8)
Supplement: Additional file 1 — Key QUERI Definitions. A glossary developed to facilitate communication and consistency within QUERI. [file 1748-5908-3-8-S1.PDF]

KEY QUERI DEFINITIONS

Best Practice [28, 59]:

- Evidence-based findings regarding an appropriate diagnostic approach, therapeutic treatment/regimen, or delivery system.
- Findings should be well established to be "best practice"; and may be found within more general evidence-based guidelines but focus on a more limited set of key clinical actions or processes.
- NOTE: QUERI is currently exploring a broader view of evidence within context of need [61-63].

Clinical best practice intervention*:

- The specific, evidence-based clinical/therapeutic practice or delivery system/organizational arrangement to be implemented to improve clinical outcomes, and for which current adherence and quality/ performance are inadequate.
- Examples are a new drug, behavioral intervention or a new clinical role or delivery system arrangement such as case management or the chronic care model.

Dissemination [59, 60]:

- An active, versus passive, effort to communicate tailored information to target audiences with the goal of engagement and information use. Dissemination is integral to implementation and is not seen as an isolated activity separate from an implementation initiative.

Facilitation [11]**:**

- Within an implementation study, a deliberate "process of interactive problem solving and support that occurs in the context of a recognized need for improvement and a supportive interpersonal relationship."

Formative evaluation [9]**:**

- "A rigorous assessment process designed to identify potential and actual influences on the progress and effectiveness of implementation efforts [p.S2, 23]."
- Conceived as occurring "before, during, and after implementation to optimize the potential for success and to better understand the nature of an initiative, need for refinements, and the worth of extending the project to other settings[p,S2-3, 23." FE is thus seen as occurring across phases within one, integrated Step 4 project. Its progressive, integrated stages within a single hybrid project are defined as developmental/diagnostic FE, implementation-focused FE, progress-related FE and interpretive FE.

Implementation*:

- Efforts designed to get best practice findings and related products into use via effective change/uptake/adoption interventions.
- Implementation typically follows dissemination and:
    - Includes identification of barriers and action steps to reduce or overcome them
    - In the presence of multiple barriers may include multiple interventions
    - Includes identification of facilitating factors and action steps to foster success.

Implementation intervention*:

- A single method or technique to facilitate change and thereby adoption of best practice recommendations. For example, an opinion leader, electronic clinical reminder, or interactive education program.
- Also referred to as "uptake" or "adoption" or "change" interventions.

Implementation strategy or program*:

- An integrated set (bundle, package) of implementation interventions. Implementation studies typically evaluate implementation strategies or programs, rather than individual interventions, in that individual interventions are rarely sufficient to achieve implementation in complex clinical settings.

**Implementation toolkit*:**<br><br>• A package of implementation interventions that is developed, tested, and refined by implementation researchers for dissemination to health care facilities for use in spreading a targeted best practice intervention or program.<br><br>• Toolkits often include educational material, data collection tools, pocket reference guides, decision-making algorithms, etc. Increasingly, additional elements essential for effective replication and sustained implementation are being considered for inclusion, e.g., methods for engaging stakeholders or assessing readiness; descriptions of the need for and role of a facilitator; barriers to/solutions for sustainability; and the approaches to and usefulness of formative evaluation before, during and after implementation.
**From internal QUERI documents, 2000-2001*
