# Supplementary material for: Overview of the VA Quality Enhancement Research Initiative (QUERI) and QUERI theme articles: QUERI Series
Source: Implement Sci. 2008 Feb 15;3:8. doi: 10.1186/1748-5908-3-8 (PMC2289837; doi:10.1186/1748-5908-3-8)
Supplement: Additional file 2 — VA QUERI Service Directed Projects: Proposal Review. Critical aspects of a proposal review checklist developed to emphasize implementation-oriented criteria. [file 1748-5908-3-8-S2.PDF]

VA QUERI Service Directed Projects: Proposal Review Criteria[1]

Adequacy of Response to Previous Reviewer Comments

- ❑ **Did the applicant address the issues raised by the reviewers of the concept paper or previously reviewed full proposal (if this is a resubmission)?**

Project Goal(s) and Questions/Hypotheses

- ❑ **Is the project's overall goal(s) described in light of the quality enhancement (QE) or performance problem(s) the project is targeting?**
- ❑ **If the project aims to address an intervening barrier or factor *contributing* to the quality problem (but not *directly causing* the quality problem), does the proposal explain how the project will contribute to the solution of the overarching quality problem, and why a direct solution is not possible?**

Background of Context

- ❑ **Does the proposal provide an adequate literature review and evidence-based clinical recommendations/guidelines or other foundations supporting the hypothesized effectiveness of the proposed quality improvement approach?**
- ❑ **Is there an adequate description of current practices, determinants, barriers and facilitators?**

Significance

- ❑ **Does the proposal adequately describe the clinical/quality issue(s) to be addressed, including as appropriate data on the clinical condition's/problem's prevalence/incidence, mortality/morbidity, quality of life consequences, economic consequences, or other significant considerations.**
- ❑ **Is the proposed work grounded in theoretical and empirical evidence on organizational change and/or provider behavior?**
- ❑ **Is the project aimed at creating a learning organization focused on the translation of research into practice?**

Methods

- ❑ **Is an overall conceptual framework for the approach provided, citing specific sources and justifying the selection of the source(s) and framework for the specific quality problem and intervention approach planned?**
- ❑ **Are the design and methods appropriate given the stated project goals?**
- ❑ **Does the work involve a clearly articulated process or formative evaluation?**
- ❑ **If an intervention is being implemented, is it adequately described (e.g., are components specified, is it apparent who will administer the intervention and to whom it is targeted) and justified?**
- ❑ **Is the overall research design, including issues such as the experimental unit (facility, clinic, team, clinician, or patient) and other major design features justified?**
- ❑ **Are the variables, measures and data collection methods/plans adequately described?**
- ❑ **Impact (summative) evaluation: overall plan**?

---

[1] The most critical points are in **bold**.

- ❑ **Are plans for identifying and recruiting all relevant participants, including clinicians, other staff (managers, support staff), patients, patient family members or caregivers, etc., discussed and any human subjects issues addressed?**
- ❑ **Is an Economic Analysis appropriate to VA decision makers (e.g., cost consequences, as opposed to traditional cost-effectiveness) included?**

<u>Adequacy of Evidence-Base Supporting Implementation *at This Time*</u>

- ❑ **Does the proposal clearly demonstrate the existence of an adequate evidence-base, in the form of published research and/or guidelines, to warrant Implementation at this time?**
- ❑ Are there known or potential risks to patients if implementation is <u>delayed</u>?
- ❑ Is it clear that implementation is not being rushed (therein creating potential patient risk)?
- ❑ Is there a critical level of need and/or urgency for implementation at this time?
- ❑ Does the implementation plan appear to target a viable system or organization (e.g., it may only be feasible to implement an intervention at a single unit – with VISN2 support – as a *step* toward implementing VAMC- and/or VISN-wide)?
- ❑ Is there sufficient supplemental evidence to support implementation if a substantial body of effectiveness data has not as yet been published (e.g., cumulative efficacy data, practical/clinical evidence, etc.)?

<u>Involvement of Key Stakeholders</u>

- ❑ **Is there evidence of commitment, including tangible resources, at all necessary levels (e.g., provider, unit, facility, VISN)?**

<u>Contribution to the Veterans Health Administration</u>

- ❑ **Does the proposed work have the clear potential to improve the quality, effectiveness and efficiency of health care in VA and the health status of veterans?**
- ❑ Does the proposed work hold the promise of rapid clinical and organizational improvement?
- ❑ **Is there evidence that the activities planned for implementation would be <u>sustainable</u> beyond the life of the proposed project?**
- ❑ Is there potential for expansion throughout VHA if the implementation is successful at the level proposed within the scope of the project (e.g., if implementation is to take pace within a single unit, is there potential for expansion throughout on or more VISN(s))?

<u>Evaluation Plan</u>

- ❑ **Does the proposal include a well-structured evaluation plan?**
- ❑ **Will spread and rollout (i.e., to other sites, clinics, VISNs) be tracked?**

<u>Dissemination/Implementation Plan</u>

- ❑ **Evaluate how and when research results will be disseminated and implemented**

---

[2] VA's decentralized, clinical delivery networks that are organized by regions with the US.
