# Supplementary material for: Overview of the VA Quality Enhancement Research Initiative (QUERI) and QUERI theme articles: QUERI Series
Source: Implement Sci. 2008 Feb 15;3:8. doi: 10.1186/1748-5908-3-8 (PMC2289837; doi:10.1186/1748-5908-3-8)
Supplement: Additional file 3 — Key Stroke QUERI Collaborators Diagram. Sample of the type of partnerships established by a QUERI Center. [file 1748-5908-3-8-S3.PDF]

**Key Stroke QUERI Collaborators (Wiring Diagram)**

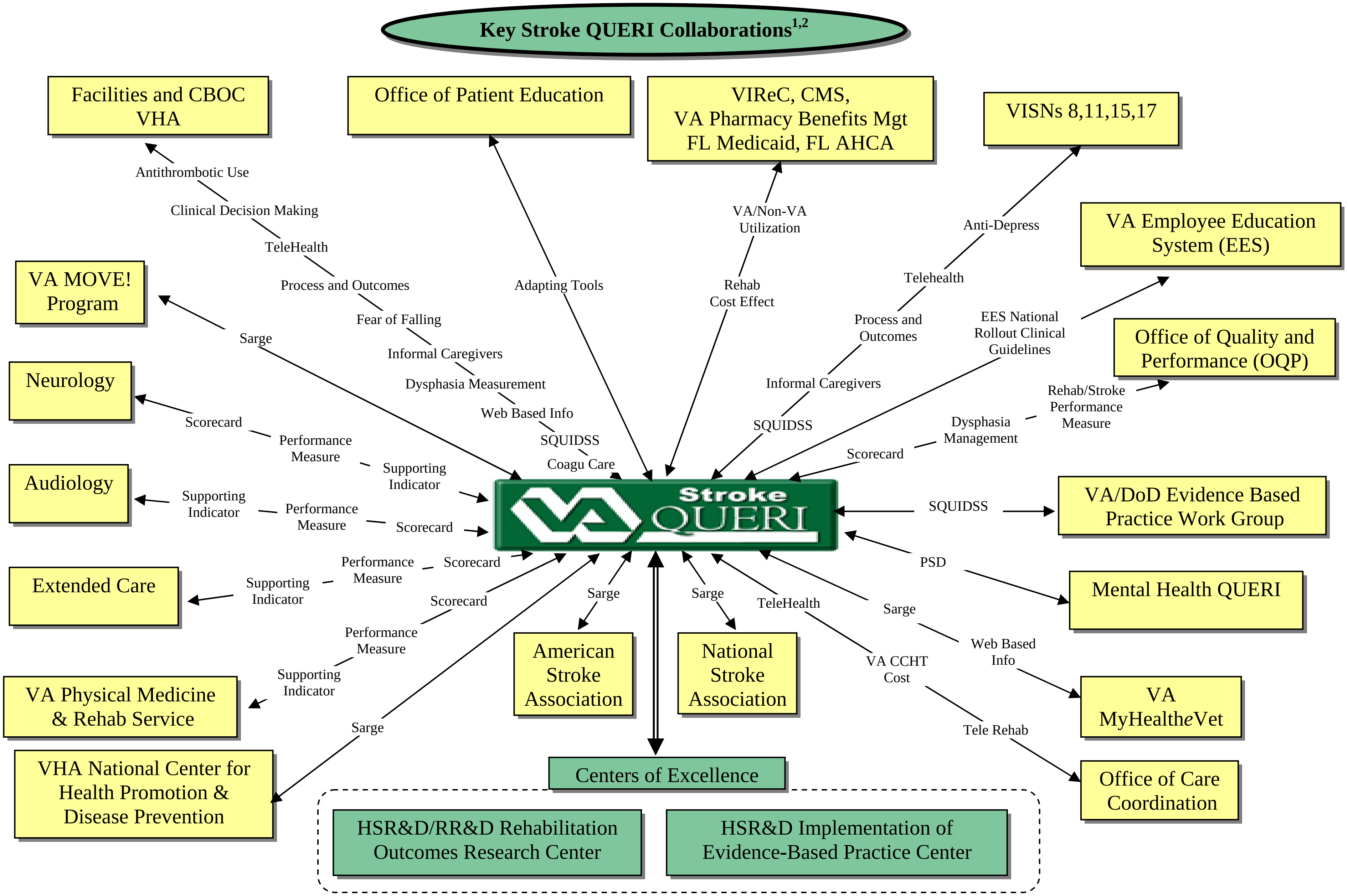


[1]Acronyms: CBOC=Community-Based Outpatient Clinics; CMS=Centers for Medicare and Medicaid Services; DoD=Department of Defense; FL AHCA=Florida Agency for Healthcare Administration; HSR&D=VA Health Services Research & Development; PSD=Post-stroke Depression; QUERI=Quality Enhancement Research Initiative; RR&D=VA Rehabilitation Research & Development; VISN=Veterans Integrated Service Network; VIReC=VA Information Resource Center.

[2]Content along arrows: This content reflects the foci of activity across various QUERI Steps. Most are self-explanatory. Those that aren't self-evident have the following meaning: MOVE!= Managing Overweight And/Or Obesity For Veterans Everywhere program; Sarge=Project titled "Disseminating Stroke Prevention Materials to Veterans: A Direct-to-Consumer Implementation Strategy"; Scorecard=Service Activity: Stroke Continuum of Care Scorecard; SQUIDSS=Project titled "Stroke Quality Improvement Decision Support System"; VA CCHT=Care Coordination/Home Telehealth program.
